# Mammographic features associated with interval breast cancers in screening programs

**DOI:** 10.1186/s13058-014-0417-7

**Published:** 2014-08-26

**Authors:** Norman F Boyd, Ella Huszti, Olga Melnichouk, Lisa J Martin, Greg Hislop, Anna Chiarelli, Martin J Yaffe, Salomon Minkin

**Affiliations:** 1 The Campbell Family Institute for Breast Cancer Research, Ontario Cancer Institute, Room 10-415, 610 University Avenue, Toronto, M5G 2M9 ON Canada; 2 School of Population and Public Health, University of British Columbia, 2206 East Mall, Vancouver, V6T 1Z9 BC Canada; 3 Prevention and Cancer Control, Cancer Care, 620 University Avenue, Toronto, M5G 2L7 ON Canada; 4 Imaging Research, Sunnybrook Health Sciences Centre, 2075 Bayview Avenue, Toronto, M4N 3M5 ON Canada; 5 Ontario Cancer Institute, 610 University Avenue, Toronto, M5G 2M9 ON Canada

## Abstract

**Introduction:**

Percent mammographic density (PMD) is associated with an increased risk of interval breast cancer in screening programs, as are younger age, pre-menopausal status, lower body mass index and hormone therapy. These factors are also associated with variations in PMD. We have examined whether these variables influence the relative frequency of interval and screen-detected breast cancer, independently or through their associations with PMD. We also examined the association of tumor size with PMD and dense and non-dense areas in screen-detected and interval breast cancers.

**Methods:**

We used data from three case-control studies nested in screened populations. Interval breast cancer was defined as invasive breast cancer detected within 12 months of a negative mammogram. We used a computer-assisted method of measuring the dense and total areas of breast tissue in the first (baseline) mammogram taken at entry to screening programs and calculated the non-dense area and PMD. We compared these mammographic features, and other risk factors at baseline, in women with screen-detected (*n*?=?718) and interval breast cancer (*n*?=?125).

**Results:**

In multi-variable analysis, the baseline characteristics of younger age, greater dense area and smaller non-dense mammographic area were significantly associated with interval breast cancer compared to screen-detected breast cancer. Compared to screen-detected breast cancers, interval cancers had a larger maximum tumor diameter within each mammographic measure.

**Conclusions:**

Age and the dense and non-dense areas in the baseline mammogram were independently associated with interval breast cancers in screening programs. These results suggest that decreased detection of cancers caused by the area of dense tissue, and more rapid growth associated with a smaller non-dense area, may both contribute to risk of interval breast cancer. Tailoring screening to individual mammographic characteristics at baseline may reduce the number of interval cancers.

**Electronic supplementary material:**

The online version of this article (doi:10.1186/s13058-014-0417-7) contains supplementary material, which is available to authorized users.

## Introduction

Screening for breast cancer with mammography is motivated by the evidence that the pre-symptomatic detection and treatment of the disease reduces mortality from breast cancer [1],[2]. Reduction of mortality from breast cancer by mammographic screening is possible because a substantial number of breast cancers have a prolonged period of time (the sojourn time) before they give rise to symptoms or signs, during which they can be detected by mammography and cured with currently available treatments [3].

Not all breast cancers, however, are detected by mammography and some are detected clinically in the interval after a negative screening mammogram. Such `interval' breast cancers have been associated with several factors including younger age, pre-menopausal status, lower body mass index (BMI), and greater mammographic density and hormone therapy [4]-[7].

In a published case-control study nested within three cohorts of women undergoing mammographic screening we found that, compared to women with less than 10% density in the mammogram, women with more than 75% density had a increased risk of breast cancer (odds ratio: 4.7; 95% confidence interval: 3.0, 7.4). For women whose breast cancer was detected at screening the odds ratio for >75% density compared to <10% was 3.5 (95% CI: 2.0, 6.2). For women in whom breast cancer was detected 12 months or less after a negative screening mammogram the odds ratio for >75% density compared to <10% was 17.8 (95% CI: 4.8, 65.9). For women in whom breast cancer was detected more than 12 months after a negative screening mammogram the odds ratio for >75% density compared to <10% was 5.7 (95% CI: 2.1, 15.5) [8].

Age, menopausal status, BMI, and hormone therapy are all associated with variations in mammographic density that may `mask' breast cancer and make detection more difficult [9]-[11]. It is not known whether these factors influence risk of interval cancers independently, or through their associations with density. The purpose of the present manuscript was to examine the association of interval breast cancer in screening programs with these other factors and with the measured components of percent density, the dense and non-dense areas in the baseline mammogram. We also examined the association of tumor size with these mammographic features in screen-detected and interval breast cancers.

Because the large increase in risk of breast cancer associated with mammographic density after a negative screening mammogram was limited to the 12 months following screening we have confined the present analysis to a comparison of two groups, women with breast cancer detected at screening and women with breast cancer detected within 12 months of a negative screening mammogram [8].

## Methods

### General method

We have carried out a study using data from three nested case-control studies carried out in populations screened with mammography. We measured mammographic density in the first (baseline) mammogram obtained at entry to each screening program with a computer-assisted quantitative method [12], and compared the baseline mammographic, demographic and other characteristics of women who developed breast cancer detected by screening with those who developed interval breast cancers. Ethical approval of the study was obtained from the University of Toronto, The University Health Network (Toronto), The Ontario Breast Screening Program, and the University of British Columbia.

### Screened populations

Selected characteristics of the screened populations included in this study are shown in Table 1. The National Breast Screening Study (NBSS) was a randomized trial of screening with mammography and physical examination [13]. The Screening Mammography Program of British Columbia (SMPBC) uses mammography as the only screening modality at the screening center, and the Ontario Breast Screening Program (OBSP) uses mammography and physical examination as screening modalities.

**Table 1:** **Selected characteristics of mammographic screening programs**

Program	National Breast Screening Study (NBSS)	British Columbia (SMPBC)	Ontario (OBSP)
**Years of operation**	1984-90	1988-present	1992-present
**Incident cancers selected for present study in years**	1984-90	1993-99	1993-98
**Number of first examinations in selected years**	45,000	250,584	166,254
**Recruitment**	Self-referral	Letter of invitation, physician and self-referral	Physician and self-referral
**Number of centers**	15	19	8
**Ages**	40-59	40-70	50-69
**Frequency of screening**	Annual	Annual	Every 2 years
**Physical examination**	Yes	No	Yes

### Selection of subjects

For the OBSP and SMPBC, lists were prepared of subjects with histologically verified invasive breast cancer diagnosed during the years 1992 to 1998 for the OBSP, and 1993 to 1999 for the SMPBC. Subjects diagnosed with breast cancer within 12 months of their first screening examination were excluded. For each case, the method of breast cancer detection, whether or not at screening, was determined by each program, independently of this study, and was based upon the active follow-up of women in whom abnormalities had been found. In addition, each program periodically carried out linkages with provincial and national cancer registries to identify breast cancers diagnosed in screened subjects in whom breast cancer had not been detected at screening.

Informed consent had been obtained at entry to the NBSS for research applications using the data collected, and all 354 subjects diagnosed with invasive breast cancer between 1984 and 1990, were included [14]. Eligible cases in the OBSP and SMPBC were sent a letter, followed by a telephone call, and asked to provide informed consent for the release of their mammogram, and to complete a self-administered questionnaire (see below). Fifty percent of cases selected from the OBSP and SMPBC agreed to take part.

### Data collection

In the NBSS, information on risk factors for breast cancer was obtained at the time of entry by self-administered questionnaire. For the other two programs, information was collected by self-administered questionnaire at the time of recruitment into the present study. Questions included demographic information, use of hormone therapy, including the date started and duration of use, as well as menstrual and reproductive risk factors, and self-reported height and weight, from which BMI was calculated. All information was collected with reference to the time of the first (baseline) screening mammogram.

Selection and/or recall bias might influence information about risk factors obtained in the OBSP and SMPBC, but not the NBSS. However, the distribution of non-mammographic risk factors was similar in all three programs and we also observed the expected effects of most known risk factors in all programs [8]. Age-adjusted BMI at entry to each screening program was correlated with percent mammographic density in the first screening mammogram in the NBSS (r?=??0.39), OBSP (r?=??0.43) and the SMPBC (r?=??0.45). In light of the similarity of results in all three programs, selection or recall bias in the OBSP and SMPBC is unlikely.

The maximum diameter of breast cancers was determined by macroscopic examination and was extracted from pathology reports obtained from the screening programs.

### Mammographic density assessment

Mammographic density was measured using a previously described computer-assisted method [12]. One cranio-caudal image was digitized for each subject, using a Lumisys 85 digitizer, and measured by one observer (NFB), in sets of approximately 120, with equal numbers of randomly ordered cases and controls. A 10% random sample of images was re-read, within and between each session, and reliability was 0.94 both within and between reads.

### Statistical methods

Of the 1,209 cases recruited, 95 were excluded because of missing data (NBSS?=?24; OBSP?=?34; SMPBC?=?37), leaving a total of 1,114 cases. Of these, 718 had been detected by screening, 125 were detected in the 12 months after a negative screening mammogram, and 271 more than 12 months after a negative screen. In the analysis, we compared the characteristics of women with breast cancer detected by screening with those with breast cancer found in the 12-month interval after a negative screen.

We used univariable logistic regression analysis to examine the associations of demographic and anthropometric variables, and mammographic measures of percent density, dense area and non-dense area with the relative frequency of interval breast cancer compared to screen-detected breast cancer. We then carried out multivariable analyses to compare features associated with the relative frequency of interval and screen-detected breast cancers, adjusting the demographic and anthropometric variables for each of the three mammographic measurements, as well as for the dense and non-dense areas together. To illustrate the magnitude and directions of the associations found, we divided percent density, dense area and non-dense area into tertiles. Although in other studies we found it necessary to apply a transformation to the three mammographic measures, in this study we present results based on the tertiles of these variables, which will not be affected by a monotonic transformation.

We use, as is common in medical research, an independent risk factor to denote a variable that has a significant contribution to an outcome in a statistical model that includes other established risk factors [15].

To examine the association between maximum tumor diameter and tertiles of percent density, dense area and non-dense area, we used multiple linear regression models adjusted for age, BMI and menopausal status that included an interaction with the cancer detection method. We compared the least square means of maximum tumor diameter obtained from these models in screen-detected and interval breast cancers within tertiles of mammographic measures, and we tested for linear trends across tertiles of mammographic measures. Tumor diameter was log transformed to improve the symmetry of the distribution and stabilize the variance, and the results are shown after back-transformation.

## Results

### Characteristics of subjects with screen-detected and interval breast cancers

Table 2 shows selected characteristics of women with breast cancer detected at screening and those in whom breast cancer was detected within 12 months of negative screening mammogram. The statistical comparisons shown were performed without adjustment for the other factors shown in the Table. In univariable analysis, younger age, lower BMI, and pre-menopausal status, a greater percent density, a greater area of dense tissue, and a smaller area of non-dense tissue, were all significantly and positively associated with breast cancer detected within 12 months of a negative screening examination. The total breast area was also associated with interval breast cancers, but this association was entirely accounted for by the dense and non-dense areas. The other variables shown in the table were not significantly associated with interval breast cancer.

**Table 2:** **Selected characteristics of screen-detected and interval cancers**

	Mean (SD)		***P***value^a^
	Screen detected N?=?718	Interval cancers N?=?125	
Age (years)	57.2 (9.0)	52.8 (8.3)	<0.0001
Body mass index (kg/m^2^)	25.3 (4.3)	23.9 (3.5)	0.0008
Age at menarche (years)	12.9 (1.5) N?=?701	12.8 (1.6) N?=?122	0.76
Parity (% parous)	84.3	82.4	0.60
Age at first live birth (years)	24.7 (4.6) N?=?605	24.7 (4.8) N?=?103	0.96
Number of live births	2.53 (1.8)	2.32 (1.6)	0.24
Menopausal status (% post-menopausal)	78.3	57.6	<0.0001
Age at menopause (years)	46.7 (6.8) N?=?496	47.1 (5.9) N?=?67	0.49
Current use of HRT^b^ (% yes)	18.3	20.8	0.50
Previous breast biopsy (% yes)	16.6 N?=?709	21.8 N?=?124	0.17
First-degree relatives with breast cancer (% yes)	20.6 N?=?714	20.0	0.88
Percent mammographic density	30.3 (19.1)	42.2 (20.4)	<0.0001
Dense area (cm^2^)	36.7 (25.6)	45.3 (28.5)	0.001
Non-dense area (cm^2^)	100.4 (60.1)	67.2 (44.6)	<0.0001
Total area (cm^2^)	137.0 (60.4)	112.5 (54.2)	<0.0001

^a^*P* value is from the univariable logistic regression model analysis of risk of interval vs. screen-detected breast cancer; ^b^hormone replacement therapy.

### Comparison of subjects with screen-detected and interval breast cancers: multivariable analysis

**Figure 1:**
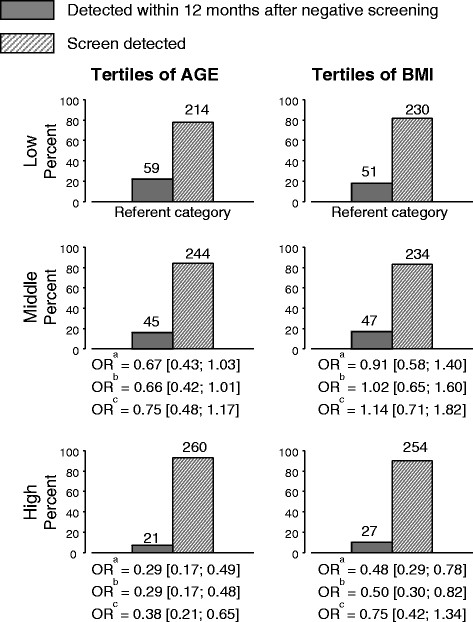
**Screen-detected and interval breast cancers according to tertiles of age and BMI at entry.** Tertiles of age: Low (39 to 52); Middle (52 to 60); High (60 to 80). Tertiles of BMI: Low (16 to 23); Middle (23 to 26); High (26 to 50). ^a^Unadjusted; ^b^mutually adjusted (age and BMI); ^c^mutually adjusted and adjusted for dense and non-dense area. BMI: body mass index; OR: odds ratio.

Figure 1 shows the associations of tertiles of age and BMI with the frequencies of screen-detected and interval breast cancers. Odds ratios for the relative frequency of interval breast cancers were calculated with reference to the lowest tertile of age and BMI and are shown before and after mutual adjustment. Menopausal status was no longer significantly associated with interval breast cancer after adjustment for age and is omitted from further analyses (data not shown).

In the lowest tertile of age (mean?=?47 years) 59 of a total of 273 cancers (22%) were detected in the 12 months after a negative screen while in the highest tertile of age (mean?=?67 years) 21 of a total of 281 cancers (7%) were interval cancers. Compared to the lowest tertile of age the highest tertile of age was associated with an odds ratio of 0.29 (95% CI: 0.17, 0.48) after adjustment for BMI indicating a significant reduction in the relative frequency of interval breast cancers with increasing age. After additional adjustment for the dense and non-dense areas of the mammogram, age remained significantly associated with a reduced relative frequency of interval breast cancer.

In the lowest tertile of BMI (mean?=?21) 51 of a total of 281 cancers (18%) were detected in the 12 months after a negative screen while in the highest tertile of BMI (mean?=?30) 27 of a total of 281 cancers (10%) were interval cancers. Compared to the lowest tertile of BMI, the highest tertile of BMI was associated with an odds ratio of 0.50 (95% CI: 0.30, 0.82) after adjustment for age, indicating a significant reduction in the relative frequency of interval breast cancer with increasing BMI. Additional adjustment for dense area alone produced similar odds ratios (data not shown), however, after additional adjustment for both the dense and non-dense areas of the mammogram, BMI was no longer significantly associated with a reduced relative frequency of interval breast cancer (OR: 0.75; 95% CI: 0.42, 1.34).

**Figure 2:**
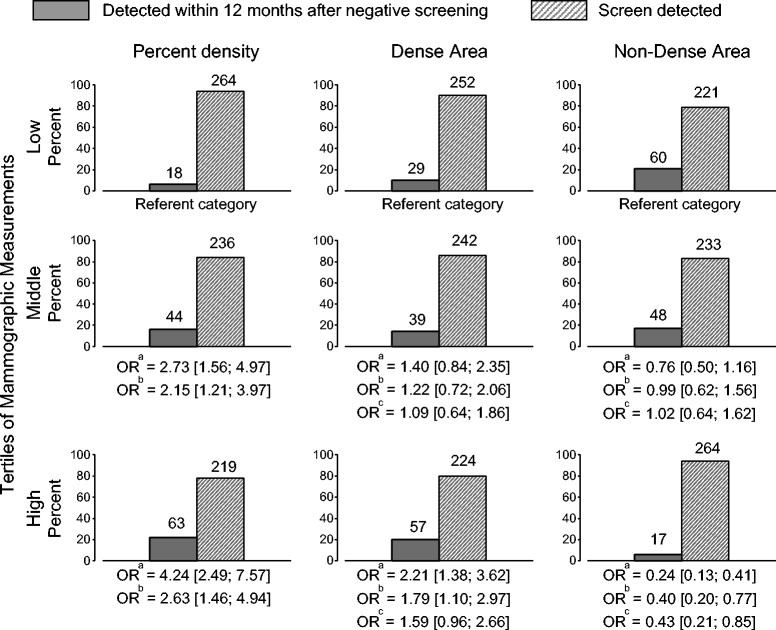
**Screen-detected and interval breast cancers according to tertiles of percent density, dense and non-dense area.** Tertiles of percent density: Low (0 to 20); Middle (20 to 41); High (41 to 84). Tertiles of dense area: Low (0 to 24); Middle (24 to 43); High (43 to 176). Tertiles of non-dense area: Low (8 to 61); Middle (61 to 112); High (112 to 344). ^a^Unadjusted; ^b^adjusted for age and BMI; ^c^mutually adjusted (dense and non-dense area) and adjusted for age and BMI. OR: odds ratio.

Figure 2 shows the associations of percent density, dense and non-dense areas with screen-detected and interval breast cancer according to the tertiles of each variable. Odds ratios for the relative frequency of interval breast cancers were calculated with reference to the lowest tertile of each mammographic measure and are shown before and after adjustment for age and BMI, and additional mutual adjustment of the dense and non-dense areas.

In the lowest tertile of percent mammographic density (PMD) (mean?=?11%) 18 of a total of 282 cancers (6%) were detected in the 12 months after a negative screen while in the highest tertile of PMD (mean?=?55%) 63 of a total of 282 cancers (22%) were interval cancers. Compared to the lowest tertile of PMD, the highest tertile of PMD was associated with an odds ratio of 2.63 (95% CI: 1.46, 4.94) after adjustment for age and BMI.

In the lowest tertile of dense area (mean?=?14 cm^2^), 29 of a total of 281 cancers (10%) were detected in the 12 months after a negative screen while in the highest tertile of dense area (mean?=?67 cm^2^) 57 of a total of 281 cancers (20%) were interval cancers. Compared to the lowest tertile of dense area, the highest tertile of dense area was associated with an odds ratio of 1.79 (95% CI: 1.79, 2.97) after adjustment for age and BMI indicating a significantly greater relative frequency of interval breast cancers with increasing dense area. After adjustment for age and BMI, and the non-dense area, the association of the dense area with interval cancers remained positive (OR?=?1.59; 95% CI: 0.96, 2.66), but was no longer statistically significant.

In the lowest tertile of non-dense area (mean?=?40 cm^2^) 60 of a total of 281 cancers (21%) were detected in the 12 months after a negative screen while in the highest tertile of age (mean?=?163 cm^2^) 17 of a total of 281 cancers (6%) were interval cancers. Compared to the lowest tertile of non-dense area, the highest tertile of non-dense area was associated with an odds ratio of 0.40 (95% CI: 0.20, 0.77) after adjustment for age and BMI, indicating a significant reduction in the relative frequency of interval breast cancers with increasing non-dense area. The non-dense area remained inversely and significantly associated with the relative frequency of interval and screen-detected breast cancers (OR?=?0.43; 95% CI: 0.21, 0.85) after additional adjustment for the dense area.

Results were unchanged when continuous measures of mammographic features were used (data not shown).

### Tumor size and mammographic measures

Table 3 shows the least square means of maximum tumor diameter for screen-detected and interval breast cancers adjusted for the other factors shown in the table footnote. Average maximum tumor diameter was greater in interval cancers than in screen-detected cancers within each tertile of each mammographic measure and, as shown in Table 3, these differences were statistically significant in most of the comparisons shown. Tests for trend in the differences in the least square means of average tumor diameter were not statistically significant over tertiles of any mammographic measures.

**Table 3:** **Average maximum tumor diameter (cms) of screen-detected and interval cancers by tertiles of mammographic measures**

Mammographic measure	Cancer detection	Least squares means (cms)^a^(95% CI) by tertiles of mammographic measures			
		Low	Middle	High	*P value* ^*b*^
Percent density	All	1.29 (1.16, 1.42) N?=?277	1.46 (1.32, 1.60) N?=?269	1.45 (1.32, 1.60) N?=?275	*0.34*
	Screen detected	1.25 (1.13, 1.39) N?=?260	1.36 (1.23, 1.51) N?=?228	1.36 (1.22, 1.51) N?=?213	*0.16*
	Interval cancers	1.50 (1.06, 2.13) N?=?17	2.02 (1.61, 2.54) N?=?41	1.87 (1.55, 2.26) N?=?62	*0.09*
	*P value* ^*c*^	*0.324*	*0.002*	*0.003*	
Dense area	All	1.33 (1.20, 1.47) N?=?275	1.35 (1.22, 1.48) N?=?273	1.50 (1.37, 1.65) N?=?273	*0.12*
	Screen detected	1.27 (1.15, 1.41) N?=?247	1.27 (1.15, 1.40) N?=?236	1.43 (1.29 1.50) N?=?218	*0.43*
	Interval cancers	1.66 (1.26, 2.19) N?=?28	1.98 (1.56, 2.52) N?=?37	1.87 (1.53, 2.27) N?=?55	*0.06*
	*P value* ^*c*^	*0.068*	*0.001*	*0.017*	
Non-dense area	All	1.39 (1.25, 1.54) N?=?276	1.45 (1.31, 1.60) N?=?271	1.35 (1.22, 1.51) N?=?274	*0.93*
	Screen detected	1.31 (1.17, 1.46) N?=?216	1.39 (1.25, 1.55) N?=?227	1.29 (1.16, 1.44) N?=?258	*0.78*
	Interval cancers	1.84 (1.51, 2.24) N?=?60	1.81 (1.45, 2.25) N?=?44	2.06 (1.43, 2.96) N?=?16	*0.78*
	*P value* ^*c*^	*0.002*	*0.031*	*0.013*	

^**a**^Least squares means were obtained from linear regression models with an interaction between cancer detection method and tertiles of mammographic measures. Tumor diameter in cms was log transformed for the analysis. Least squares means and their 95% confidence intervals (CIs) are shown transformed back to the original scale. For N?=?15 subjects with recorded tumor size?=?0, the minimum recorded tumor size?=?0.1 cm was used in the analysis. For N?=?17 subjects with screen detected, and for N?=?5 subjects with interval breast cancer the tumor diameter was not available. For tertiles of percent mammographic density, the model was adjusted for age, body mass index, and menopausal status. For tertiles of dense area, the model was, in addition, adjusted for non-dense area (continuous), and for tertiles of non-dense area, the model was adjusted for dense area (continuous). ^b^*P* values for the test for linear trend across tertiles of mammographic measurements. ^c^*P* values for comparing least square means of maximum tumor diameter in screen-detected and interval breast cancers within tertiles of mammographic measures. The *P* values were adjusted for multiple comparisons (the Tukey-Kramer adjustment).

In all cancers, after adjustment for age, BMI and menopausal status, the dense area was significantly and positively associated with maximum tumor diameter when both were treated as continuous variables (*P*?=?0.04).

## Discussion

An improved understanding of the factors that influence the frequency of interval breast cancers in screening programs may be useful in the selection of the methods and the frequency with which women are screened. Breast cancers diagnosed in the 12 months after a negative screening mammogram, referred to here as `interval cancers', might occur for several reasons. Tumors might have been present but not detected on the previous screening examination and failure of detection might be due to radiological or technical error, or because the signs of the tumor were `masked' by dense breast tissue. Alternatively, some cancers might have been present but too small to be detected by mammography, and grow to become clinically detectable in the interval before the next screen. Cancers that behave in this way are likely to have a greater than average rate of growth and a shorter than average sojourn time.

The factors previously described as associated with `interval' cancers in screening programs include younger age, lower weight or BMI, pre-menopause status and use of hormone therapy, and mammographic density. Younger age, lower weight, pre-menopausal status [9],[10],[16] and use of combined hormone therapy [11],[17]-[19] are all known to be associated with a greater area of mammographically dense breast tissue. With the exception of hormone therapy, these factors are all shown here in Canadian screening programs to be associated with a greater relative frequency of interval breast cancer compared to screen-detected breast cancer. We have shown in a separate study that the effect of hormone therapy use on risk of interval cancers is also independent of mammographic density [20].

As we show here, PMD and its constituent elements, the dense and non-dense areas of the mammogram, were each associated with risk of interval breast cancer. The relative frequency of interval breast cancer increased with greater percent density and area of dense tissue and decreased with a greater area of non-dense tissue. Adjustment for these mammographic measures rendered non-significant the previously significant association of BMI with interval breast cancer. Younger age however remained significantly and independently associated with an increased relative frequency of interval cancer after all adjustments. After adjustment, greater percent density and smaller non-dense areas of the mammogram also remained significantly associated with the frequency of interval cancers.

Although younger age is known to be associated with greater PMD [9],[10], the associations of age with the frequency of interval breast cancer was independent of the mammographic measures analyzed. Age may thus influence the frequency of interval cancers in part by an influence on the biological behavior of tumors, rather than by masking due to greater density. Interval cancers have been shown to have biological characteristics including a higher proportion of proliferating cells and more frequent expression of p53 [21] that suggest more rapid tumor growth. Also consistent with more rapid growth was the finding that interval cancers had a larger maximum tumor diameter within each tertile of the mammographic measures examined. However, in contrast to some previous studies, we did not find an association between tumor size and percent density in screen-detected or interval breast cancers [22]. Greater hormonal stimulation of breast cell proliferation and tumor growth are potential mechanisms by which younger age might influence the frequency of interval breast cancers.

The additional and independent effect of a greater BMI-adjusted non-dense area in reducing the relative frequency of interval breast cancer may also be the result of biological factors that influence tumor growth. The non-dense tissue in the adult breast is composed of adipose tissue and the lipid-laden adipocytes that form this tissue arise from the differentiation of stromal mesenchymal cells. Aromatase activity in the breast provides a source of estrogen production that may stimulate tumor growth. As reviewed in Simpson *et al*. [23] adipose tissue aromatase is expressed primarily in stromal mesenchymal cells, rather than in lipid-laden adipocytes, and aromatase in adipose tissue is thus a marker of the undifferentiated adipose mesenchymal cell. Aromatase activity in stromal mesenchymal cells diminishes with differentiation to mature adipocytes [23],[24]. The reduction of this source of estrogen after adipocyte differentiation might contribute to the inverse association observed between the area of non-dense tissue on mammography and the frequency of interval breast cancer.

Strengths of the present study include the relatively large number of breast cancers from three screening programs in Canada. Each program had classified tumors as having been detected by screening or by other means, and the method of detection played no role in the sampling of subjects for the present study and was unknown at the time mammographic measurements were made. We used a computer-assisted method of measuring mammographic features that has been shown to give measurements that are strongly and reproducibly associated with breast cancer risk. Further, we had a complete set of potentially relevant covariates that were included in the analysis.

Limitations of the study include our exclusive reliance on film rather than digital images. However, a population-based study carried out in the Norwegian Breast Cancer Screening Program by Hofvind *et al*. compared the performance of screen-film mammography (SFM) and full-field digital mammography (FFDM) in women aged 50 to 69 years. The rates of invasive interval breast cancer were 2220/1,391,188 (0.17/1000) with SFM and 309/446,172 (0.12/1000) with FFDM, a difference that was not statistically significant (*P*?=?0.07) [17].

The location of tumors in the breast and other aspects of the phenotype of the breast cancers were also not available to us. Domingo *et al*. have shown that aspects of the tumor phenotype, including HER2-positive and triple-negative tumors, and breast density are independently associated with interval breast cancers [25].

The results of the present study suggest that mammographic screening with a single imaging modality and a fixed screening interval for all women may not be optimal. Several independent sources of variation in the detection of breast cancer by screening are identified that are personal characteristics of individual women present at the time screening was initiated. The incorporation of these factors into the design and execution of screening programs for breast cancer might improve the outcomes of screening, extend the ages at which screening is carried out, and further reduce mortality from breast cancer.

In addition to digital mammography, that has now largely replaced film [26], alternative modalities include ultrasound [27], and magnetic resonance imaging [28]. Although it is known that each of these modalities can detect some breast cancers that are not seen on film mammography, their value as alternatives to mammography in the context of screening is largely unknown. Modulation of the screening interval according to characteristics that influence risk of interval cancers might also be considered, with shorter intervals for younger women with extensive dense breast tissue and longer intervals for older women with radiolucent breasts on mammography.

## Conclusions

Compared to women with screen-detected breast cancer, younger age and a greater dense area and smaller non-dense areas in the baseline mammogram were independently associated with a greater frequency of interval breast cancers in screening programs. These results suggest that decreased detection of cancers caused by the area of dense tissue, and more rapid growth associated with a smaller non-dense area, may both contribute to the frequency of interval breast cancers.

## Authors' contributions

NFB, LJM, SM with GH and AC conceived and executed the present work and with EH wrote the manuscript. EH and OM carried out the statistical analysis and prepared the graphs. GH was responsible for recruitment and the acquisition of data from the screening project in British Columbia. AC was responsible for recruitment and the acquisition of data from the screening project in Ontario. MJY developed and supplied the computer-assisted method of measuring mammographic features. All authors made substantial contributions to the interpretation of the data and critically appraised the draft manuscript, gave final approval to the version to be published, and agree to be accountable for the accuracy and integrity of the work.

## Authors’ original submitted files for images

Below are the links to the authors’ original submitted files for images. Authors’ original file for figure 1 Authors’ original file for figure 2

## Abbreviations

BMI: body mass index
FFDM: full-field digital mammography
NBSS: National Breast Screening Study
OBSP: Ontario Breast Screening Program
PMD: percent mammographic density
SFM: screen-film mammography
SMPBC: Screening Mammography Program of British Columbia
