# Supplementary figures and images for: Mammographic features associated with interval breast cancers in screening programs
Source: Breast Cancer Res. 2014 Aug 26;16:417. doi: 10.1186/s13058-014-0417-7 (PMC4187338; doi:10.1186/s13058-014-0417-7)

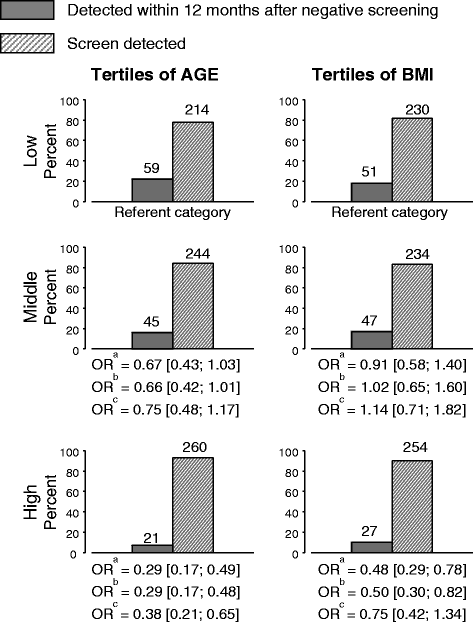

Supplement: Supplementary file 1 — Authors’ original file for figure 1 [file 13058_2014_417_MOESM1_ESM.gif]

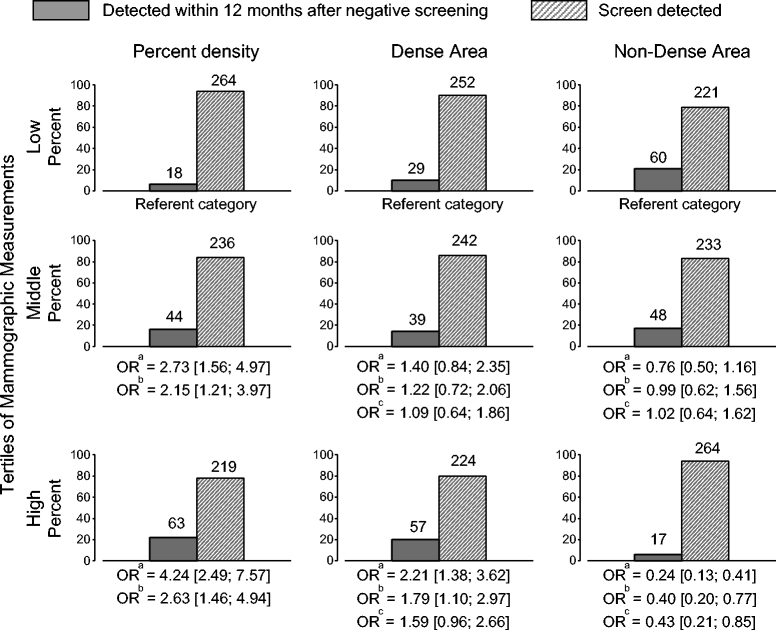

Supplement: Supplementary file 2 — Authors’ original file for figure 2 [file 13058_2014_417_MOESM2_ESM.gif]
